# Risk Factors Associated with Groin Pain in Athletes: A Systematic Review

**DOI:** 10.3390/life15111688

**Published:** 2025-10-30

**Authors:** Tamiris Beppler Martins, Taís Beppler Martins, Filippo Migliorini, Nicola Maffulli, Rodrigo Okubo

**Affiliations:** 1 Graduate Program in Physical Therapy, Universidade do Estado de Santa Catarina (UDESC), Florianopolis 88080-350, SC, Brazil; tamiris.martins@udesc.br (T.B.M.); taisbppmartins@gmail.com (T.B.M.); rodrigo.okubo@udesc.br (R.O.); 2 Department of Physical Therapy, Universidade do Estado de Santa Catarina (UDESC), Florianopolis 88080-350, SC, Brazil; 3 Department of Trauma and Reconstructive Surgery, University Hospital of Halle, Martin-Luther University Halle-Wittenberg, 06097 Halle, Germany; filippo.migliorini@uk-halle.de; 4 Department of Orthopaedic and Trauma Surgery, Academic Hospital of Bolzano (SABES-ASDAA), Via Lorenz Böhler 5, 39100 Bolzano, Italy; 5 Department of Life Sciences, Health, and Health Professions, Link Campus University, Via del Casale di San Pio V, 00165 Rome, Italy; 6 Faculty of Medicine and Psychology, University “La Sapienza” of Rome, 04019 Terracina, Italy; 7 School of Pharmacy and Bioengineering, Keele University Faculty of Medicine, Stoke on Trent ST4 7QB, UK; 8 Centre for Sports and Exercise Medicine, Barts and the London School of Medicine and Dentistry, Queen Mary University of London, Mile End Hospital, 275 Bancroft Road, London E1 4DG, UK

**Keywords:** groin pain, athletes, risk factors, hip adduction strength, injury prevention

## Abstract

Groin pain is a common and multifactorial condition in athletes, leading to performance impairment and a reduction in participation in sports. This systematic review aimed to identify and synthesise risk factors for groin pain in athletes. A comprehensive search of PubMed, Embase, Web of Science, Scopus, and SPORTDiscus was conducted from inception to May 2025. Observational studies were included, and the risk of bias was assessed using the ROBINS-E tool. Due to heterogeneity across studies, a narrative synthesis was performed. Eight retrospective cohort studies comprising 4249 male athletes from various sports met the inclusion criteria. The most consistent risk factors were a previous history of groin injury, reduced eccentric hip adduction strength, limited hip rotation, and inadequate preseason conditioning. Additional contributors included participation in Olympic weightlifting as part of sport-specific conditioning, playing in skill-specific positions, and the presence of subclinical symptoms, with associations ranging from moderate to high. The overall quality of evidence was low to moderate, with confounding and outcome measurement being the most frequent sources of bias. These findings highlight the multifactorial nature of groin pain and underscore the need for individualised screening, early detection, and preventive approaches. Future research should prioritise prospective designs, standardised diagnostic criteria, and inclusion of female athletes to improve clinical applicability.

## 1. Introduction

Groin pain is a common and multifactorial condition in athletes, especially those involved in sports that require frequent acceleration, deceleration, and repetitive twisting movements, such as football, rugby, and hockey [1,2,3]. Clinically, it presents as chronic discomfort or pain in the groin region, often exacerbated by specific sports activities [4]. This condition can lead to considerable functional limitations, prolonged time away from training or competition, and, in severe cases, early retirement from athletic careers [5,6,7].

Several intrinsic factors have been proposed as contributing to the development of groin pain, including a history of previous injury, older age, higher body mass, early biological maturation, reduced femoral head diameter in the dominant limb, limited hip range of motion—particularly in abduction and rotation—and strength imbalances between lower-limb muscle groups [8]. Among these, weakness of the adductor muscles has consistently emerged as a key intrinsic risk factor [9]. In contrast, extrinsic factors encompass variables related to sport participation and training environment, such as excessive training volume, inadequate periodisation, insufficient preseason conditioning, and the absence of structured preventive programmes [10,11,12]. These external demands may exacerbate underlying intrinsic vulnerabilities and contribute to the onset of groin pain.

Although limitations in hip internal rotation have frequently been reported [8], current evidence remains insufficient to establish definitive conclusions [13]. A previous systematic review and meta-analysis identified significant differences between athletes with and without groin pain, including lower strength in the adductor squeeze test, reduced range of motion in internal rotation and the bent-knee fall-out test, worse self-reported function, and altered trunk mechanics [14]. However, that review only included studies published between 2001 and 2014, highlighting the need for updated evidence in light of new research and clinical insights.

Despite advances in understanding groin pain, important gaps remain in the literature, including limited sample sizes, the absence of longitudinal follow-up, and methodological limitations in distinguishing among clinical subtypes, such as adductor-, iliopsoas-, inguinal canal-, or pubic-related groin pain. Accordingly, this systematic review aims to identify specific risk factors associated with groin pain in athletes, focusing on prospective and observational studies published through 2025. Quantitative variables of interest included adductor strength, sex, pain history, and engagement in preventive strategies, which were selected based on their consistent relevance in previous literature and their potential contribution to the multifactorial aetiology of groin pain. Adductor strength has repeatedly been identified as a key determinant of injury risk due to its central role in stabilising the pelvis and counteracting high eccentric loads during directional changes [9,14,15]. Sex was considered because anatomical and hormonal differences may influence muscle performance and susceptibility to injury [16]. A history of pain or previous injury represents residual neuromuscular deficits or tissue vulnerability that increases the likelihood of recurrence [8,17]. Finally, engagement in preventive strategies, such as specific strengthening or flexibility programmes, was included since these interventions may mitigate both intrinsic and extrinsic risk factors and ultimately reduce injury incidence [18,19].

The expected outcomes of this review include the identification of clinically relevant risk factors that can improve functional screening and support individualised preventive interventions. Scientifically, the review aims to map current knowledge gaps, encourage studies with more robust methodological designs, and clarify risk mechanisms underlying groin pain in athletes. From a broader perspective, the findings may contribute to long-term athletic health by reducing time-loss injuries and promoting sustained participation in sports among both youth and professional athletes. Therefore, the objective of this review is to identify and analyse the risk factors associated with groin pain in athletes, based on the most current available evidence.

## 2. Materials and Methods

This systematic review was conducted in accordance with the Preferred Reporting Items for Systematic Reviews and Meta-Analyses literature search extension (PRISMA-S) [20]. It was prospectively registered in the International Prospective Register of Systematic Reviews (PROSPERO) under the ID CRD 42022363350.

### 2.1. Search Strategy

A comprehensive search was conducted across five electronic databases: PubMed (National Library of Medicine and National Institutes of Health), Embase, Web of Science, Scopus, and SportDiscus, from their inception to May 2025. Only peer-reviewed original studies published in English that investigated risk factors associated with the development of groin pain were considered eligible. The complete search strategy and keywords used are presented in Table 1. The search terms were selected to encompass both groin- and hip-related conditions that share overlapping clinical features, ensuring comprehensive coverage of musculoskeletal sources of groin pain.

The search and selection processes were conducted independently by two reviewers (TBM and TBM). Any discrepancies were resolved by a third reviewer (RO).

### 2.2. Eligibility Criteria

Studies were included based on predefined inclusion and exclusion criteria guided by the PECOS framework (Population, Exposure, Comparison, Outcome, and Study design) [21]. A detailed summary of the criteria is presented in Table 2.

### 2.3. Study Selection and Data Extraction

Reference management and screening were performed using Rayyan software (Rayyan Systems Inc., Doha, Qatar; version 1.0.0) [22]. Two reviewers (TBM and TBM) independently conducted study selection and data extraction to reduce bias and ensure methodological rigour. Discrepancies were resolved by consensus.

The following data were extracted from each included study: (1) authors and year of publication; (2) country of the first author’s institutional affiliation; (3) study design; (4) participant characteristics (sample size, age, sex, body mass index, level of sports participation); (5) sport type; (6) follow-up period; (7) definition of groin pain; (8) primary anatomical location and diagnosis or injury; (9) reported risk factors (identified through bivariate or multivariate analyses with effect measures above 1.0); and (10) corresponding effect sizes (Odds Ratios [OR], Risk Ratios [RR], or Hazard Ratios [HR]) with 95% confidence intervals.

Identified risk factors were categorised into predefined domains based on previous literature, including individual attributes, sport- and training-related variables, health and lifestyle factors, morphological characteristics, and biomechanical parameters. This classification allowed a comprehensive overview of the variables potentially associated with groin pain in athletes.

### 2.4. Risk of Bias Assessment in Individual Studies

The methodological quality of the included studies was independently assessed by two reviewers (TBM and TBM) using the Risk of Bias in Non-randomised Studies–of Exposure (ROBINS-E) tool [23]. This tool evaluates seven domains of bias: (1) confounding, (2) exposure measurement, (3) participant selection, (4) post-exposure interventions, (5) missing data, (6) outcome measurement, and (7) selective reporting of results.

### 2.5. Data Synthesis

Given substantial heterogeneity among studies, regarding sample characteristics, definitions of exposure and outcome, and statistical methods, a meta-analysis was not feasible. Therefore, a narrative synthesis was conducted. A summary table was developed to present an overview of the methodological features, risk of bias assessments, and ROBINS-E domain ratings for each included study.

## 3. Results

**Figure 1:**
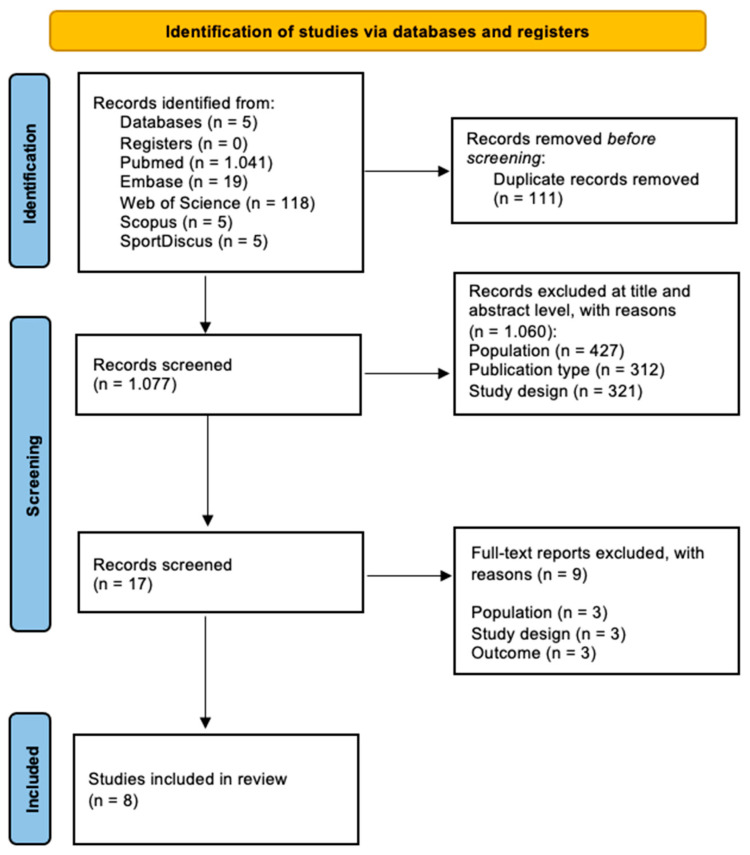
Study flowchart following PRISMA guidelines.

The initial search identified 1188 records. After removing 111 duplicates, 1077 studies were screened by title and abstract. Of these, 17 full texts were assessed for eligibility, and eight retrospective cohort studies met the inclusion criteria (Figure 1).

### 3.1. Study Characteristics

The eight included studies involved 4249 male athletes participating in a range of sports, including soccer, ice hockey, Gaelic football, and American football. The studies were conducted in the United States, Sweden, Qatar, Ireland, Denmark, Australia, and Canada, with follow-up durations ranging from one season to ten years (Table 3). Although the inclusion criteria allowed for all sport disciplines, the studies that met eligibility requirements primarily involved field- and ice-based team sports, reflecting the current distribution of available evidence.

Participants ranged from young adults to elite-level athletes competing at youth to elite levels. All studies employed observational designs and defined groin pain using clinical criteria; some also included imaging confirmation (e.g., MRI). The most frequently reported anatomical regions involved were the adductor muscles, pubic symphysis, and abdominal wall.

### 3.2. Definitions and Outcome Criteria

Groin pain definitions varied across studies. Some relied solely on clinical examination or symptom duration, while others combined clinical evaluation with imaging or standardised diagnostic criteria. Injury duration thresholds ranged from two to over six weeks of restricted participation. Diagnoses included adductor muscle strain, chronic groin pain, athletic pubalgia, and groin-related injuries without radiological findings.

### 3.3. Identified Risk Factors

Across the included studies, several risk factors were consistently associated with groin pain:

- Previous history of groin pain or injury [14,17,24].
- Reduced hip adduction strength, especially in eccentric contraction [14,25].
- Inadequate preseason conditioning or preparation [24].
- Limited range of motion in hip internal or external rotation [26,27].
- Participation in Olympic weightlifting-style training, particularly when implemented as part of strength and conditioning programmes for field-based athletes, was identified as a potential contributor to athletic pubalgia in collegiate American football players [28].
- Pain during adductor squeeze testing [25].
- Presence of subclinical groin symptoms, defined as mild discomfort, stiffness, or transient groin pain during activity that does not cause time-loss or require medical consultation, but may precede clinically evident injury [29].

**Table 3:** Included studies and key characteristics.

Reference; Study Design; Country of the Main Author	Participant Characteristics	Type of Sport	Follow-up Duration	Definition of Groin Pain	Main Anatomical Site of Injury; Primary Diagnosis/Injury	Related Risk Factors	Risk Measures (OR/RR/HR)	95% CI	Effect Size
Neuville et al., 2023 [28]; Retrospective cohort; USA	1154 male collegiate athletes; mean age not specified; BMI varied by playing position	American football	10 years (2010–2019)	Clinical diagnosis + MRI confirmation of rectus abdominis or adductor aponeurosis lesion	Pubic region (pubic symphysis); Athletic Pubalgia	OWL; skill position	OWL: OR = 2.86; Skill position: OR = 9.32	OWL: 1.25–7.35; Skill position: 1.71–63.96	Not directly reported; magnitude inferred from ORs
Wörner, Thorborg, Clarsen, and Eek, 2022 [29]; Retrospective cohort; Sweden	163 male athletes; mean age 22.6 years; 17 years of experience	Ice hockey (professional and semi-professional)	1 season (2017–2018)	Any hip or groin pain affecting participation, performance, or causing time-loss	Hip and groin; Groin-related problems including pubalgia	History of groin problems without time-loss in previous season	All problems: OR = 3.3; Substantial: OR = 3.6; Time-loss: OR = 2.3	1.7–6.3; 1.8–8.4; 0.9–5.7	Not directly reported; magnitude inferred from ORs
Mosler et al., 2018 [14]; Retrospective cohort; Qatar	438 professional male athletes	Soccer	2 seasons (~2 years)	Groin/hip injuries causing ≥1 day of time-loss from training or match	Hip and groin; Adductor-related injuries	Previous injury history; reduced eccentric adduction strength	Prior injuries: HR = 1.8; Eccentric strength: HR = 1.6	1.2–2.7; 1.0–2.5	Not directly reported
Delahunt, Fitzpatrick, and Blake, 2017 [25]; Retrospective cohort; Ireland	55 professional athletes; 24 ± 2.8 years; BMI 24.7 kg/m^2^	Gaelic Football	1 season	Groin injury causing time-loss from training or matches	Hip and groin; Groin injury	Adductor squeeze < 225 mmHg; HAGOS < 87.5; Squeeze pain > 0	OR = 7.78; OR = 8.94; OR = 2.16 per pain point	Not directly reported	Not directly reported
Hölmich et al., 2014 [17]; Retrospective cohort; Denmark	998 male sub-elite athletes; mean age not specified	Sub-elite soccer	10 months (1 season)	Clinically diagnosed groin injuries (standardised criteria)	Adductor, iliopsoas, abdominal region; Adductor, iliopsoas and abdominal injuries	Age and history of previous groin injury	HR = 2.13 (previous injury); RIT = 2.28 (adductor); RIT = 4.56 (adductor + abdominal)	1.23–3.67; 1.22–4.25; 1.91–10.91	Significantly longer recovery time
Ibrahim, Murrell, and Knapman, 2007 [26]; Retrospective cohort; Australia	120 professional athletes; mean age not specified	Soccer	1 season	Clinically diagnosed adductor muscle injuries	Adductors; Adductor injury	Reduced hip range of motion	Not directly reported	0.88 (internal rotation) and 0.96 (external rotation)	Not directly reported
Verrall et al., 2007 [27]; Retrospective cohort; Australia	29 professional athletes; mean age not specified	Soccer	1 season	Chronic groin pain >6 weeks diagnosed by clinical criteria and time-loss	Hip; Chronic groin injury	Reduced hip ROM before injury	Incident rate ratio: Weight (0.92) Total ROM (0.90)	Weight (0.871–0.974) Total ROM (0.834–0.992)	Not directly reported
Emery et al., 2001 [24]; Retrospective cohort; Canada	1292 male athletes; mean age not specified	Ice hockey (NHL)	Not specified	Clinically diagnosed adductor or abdominal muscle injuries	Adductor or abdominal region; Adductor or abdominal muscle injury	Low preseason sport-specific training; previous injury; NHL experience	RR = 3.38 (low training); RR = 2.88 (previous injury); RR = 5.69 (veterans)	1.45–7.92; 1.33–6.26; 2.05–15.85	Not directly reported

Caption: CI = confidence interval; BMI = body mass index; kg/m^2^ = kilograms per square metre; MRI = magnetic resonance imaging; ROM = range of motion; NHL = national hockey league; OWL = Olympic weightlifting; OR = odds ratio; RR = relative risk; HR = hazard ratio; RIT = relative injury time.

### 3.4. Measures of Association and Effect Size

When available, statistical estimates revealed moderate to high associations between risk factors and groin pain:

- Olympic weightlifting increased injury odds (OR = 2.86; 95% CI: 1.03–7.96), with skill-position players showing even higher risk (OR = 9.32; 95% CI: 1.80–48.27) [28].
- Inadequate preseason fitness was associated with increased risk (RR = 3.38; 95% CI: 1.04–10.97) [24].
- Previous groin pain predicted future injury (HR = 2.13; 95% CI: 1.07–4.26) [17].
- Pain during the adductor squeeze test was linked to injury occurrence (OR = 1.46; 95% CI: 1.01–2.11) [25].

Although not all studies reported effect sizes, consistent patterns emerged linking prior symptoms and physical impairments to the development of injury.

### 3.5. Synthesis Overview

Overall, risk factors for groin pain in athletes appear to be multifactorial, involving individual characteristics, physical capacity, symptom history, and sport-specific demands. The findings emphasise the importance of preseason screening and the implementation of targeted prevention strategies, particularly focusing on strength, flexibility, and symptom monitoring.

### 3.6. Quality and Risk of Bias Assessment

**Figure 2:**
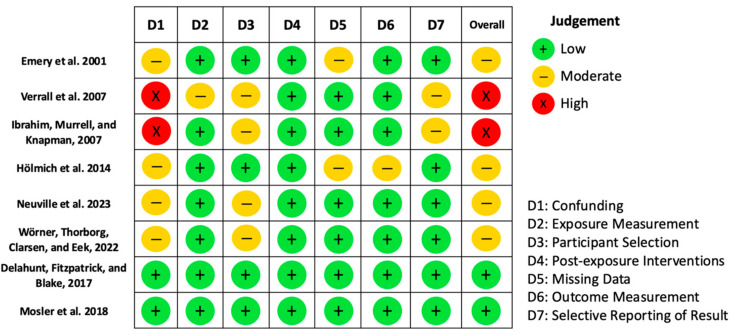
Summary of risk of bias assessed using the ROBINS-E tool [14,17,24,25,26,27,28,29].

Methodological quality and risk of bias are summarised in Table 4 and illustrated in Figure 2. Sample sizes ranged from small, homogeneous cohorts [14,25] to large, representative populations [20,21]. Most studies employed validated and reliable tools to assess exposure variables, including hip range of motion, strength, and training volume. However, repeated measures and formal reliability reporting were frequently absent.

Confounding was inconsistently addressed: while some studies adjusted for key variables (e.g., prior injury, training load, position), others did not, resulting in a moderate to high risk of bias in this domain. Missing data were generally low or well-managed. Outcome assessment was primarily based on clinical diagnosis or structured surveillance systems, although a few studies relied on self-reported symptoms, which may have reduced diagnostic accuracy.

Reporting bias was considered low, with transparent presentation of results in most studies. None of the included studies implemented post-exposure interventions, thereby minimising the risk of bias from treatment effects. According to the ROBINS-E tool, the overall risk of bias ranged from low to moderate, with the domains of confounding and outcome measurement being the most frequently affected. These limitations should be taken into account when interpreting the strength of the reported associations.

## 4. Discussion

This systematic review identified and synthesised risk factors associated with groin pain in athletes. Across the eight included retrospective cohort studies, the most consistently reported risk factors were a prior history of groin pain, reduced hip adduction strength, particularly during eccentric contractions, limited hip rotation range of motion, and insufficient preseason conditioning. Additionally, specific sport-related exposures, such as participation in Olympic weightlifting-style training and playing in skill positions, were associated with an increased risk of injury. In this context, Olympic weightlifting-style exercises are commonly incorporated into strength and conditioning routines across field-based sports to enhance power production; however, the associated high-load hip extension and stabilisation demands may contribute to increased groin stress when not properly monitored. In this review, the mention of Olympic weightlifting refers to its role as a conditioning exposure integrated into team-sport training, rather than as a standalone sport. These findings emphasise the multifactorial nature of groin pain, which involves the interplay between individual impairments, symptom history, and sport-specific demands.

Among the identified factors, previous groin injury was the most consistently reported across studies [14,17], corroborating broader sports injury literature, where injury history is a well-established predictor of recurrence due to residual neuromuscular deficits or suboptimal rehabilitation [8,30]. This finding should be interpreted with caution, as a previous episode of groin pain may reflect residual tissue vulnerability or incomplete recovery rather than a direct causal relationship. In this sense, previous pain acts as an indicator of increased susceptibility to recurrence, rather than an isolated etiological factor. A recent meta-analysis also found significantly lower adduction strength in athletes who developed groin pain compared to those who did not (standardised mean difference = −0.5) [15].

Reduced eccentric adduction strength was another notable finding, as highlighted in several studies [25]. Further evidence supported this, reporting significantly lower eccentric strength in symptomatic athletes (2.47 vs. 3.12 Nm/kg), while isometric strength did not differ [31]. These data emphasise the clinical utility of eccentric strength tests, such as the 45° adductor squeeze test, for screening and prevention [32]. However, it is essential to acknowledge that the number of studies supporting each identified risk factor remains limited, and most available evidence derives from studies of moderate methodological quality. This limitation should be considered when interpreting the strength of the associations presented in this review. Taken together, these findings suggest that groin pain is not driven by a single deficit but rather by an interaction between mechanical load, neuromuscular capacity, and tissue tolerance. Specifically, inadequate adductor strength may reduce the capacity to counteract high eccentric loads during directional changes and kicking, thereby increasing tensile stress on the adductor–abdominal aponeurosis and pubic symphysis. Limitations in hip rotation and suboptimal preseason conditioning may further compromise load distribution. These interdependent mechanisms provide a plausible explanation for the multifactorial aetiology consistently described in the literature and support the need for integrated prevention strategies rather than isolated strength-based approaches.

Evidence from youth populations also supports the role of eccentric strength. The Copenhagen Adduction Exercise has been shown to significantly increase eccentric strength and reduce the risk of adductor-related groin pain, with a mean increase of 0.49 Nm/kg (*p* < 0.00001) [18]. However, even with preventive programmes, other modifiable factors such as abduction ROM and dominant-leg strength imbalances may persist, highlighting the need for individualised protocols across age groups [19].

Limitations in hip rotation range of motion were also associated with an increased risk of injury [26,27]. These impairments may reflect capsular restrictions or early degenerative changes, altering pelvic load distribution. While not independent predictors, ROM restrictions may exacerbate risk when combined with strength deficits or inadequate conditioning. The broader literature supports moderate evidence for ROM loss following the onset of groin pain [32].

Inadequate preseason conditioning was identified as a modifiable risk factor, with an increased incidence of injuries among less conditioned athletes [24], reinforcing the need for structured preparation periods. Specific exposures, such as Olympic weightlifting-style training and playing in skill positions, also increased the odds of injury [28], possibly due to higher load demands or explosive actions.

Subclinical symptoms were another relevant finding. Authors demonstrated that athletes with early, non-disabling groin symptoms had a greater likelihood of developing injuries, emphasising the importance of symptom surveillance and early intervention to prevent chronicity [29].

The included studies presented moderate methodological quality, with frequent limitations in confounding control, such as age, level of play, and prior injuries. Most studies used valid tools for exposure measurement, but varied in protocol standardisation and examiner reliability. According to the ROBINS-E, confounding and outcome measurement were the main sources of bias, which limit causal inferences. Despite these limitations, the present review provides value by systematically summarising the current evidence base and identifying recurrent methodological gaps that have hindered progress in this area. By outlining the most consistent associations and highlighting the need for standardised diagnostic and analytical approaches, this work contributes to refining research priorities and improving the design of future studies on groin pain in athletes.

All included studies employed retrospective cohort designs, inherently limiting causal inference and temporal relationships. Additionally, substantial heterogeneity in diagnostic criteria and outcome definitions restricted direct comparisons across studies. While some relied solely on clinical assessment, others incorporated symptom duration or imaging findings, reducing consistency and generalizability. These methodological limitations are consistent with the previous literature, which indicates low overall evidence quality in groin injury research. For example, a systematic review of groin pain treatments in athletes reported that only 6% of studies were rated as high quality, with lower-quality studies more frequently reporting positive outcomes [33]. These findings underscore the urgent need for rigorous study designs, standardised diagnostic protocols, and consistent outcome measures to enable robust clinical conclusions.

Our findings are consistent with those of the systematic review, which also identified previous groin injury and reduced adductor strength as the most consistent risk factors [8]. However, our review expands upon their work by incorporating more recent evidence (up to 2025), including studies on subclinical symptoms, youth populations, and sport-specific demands such as Olympic weightlifting and playing positions. Additionally, our review addresses persistent gaps in terminology standardisation and female athlete representation, which were not emphasised in earlier reviews. These advancements reinforce the need for ongoing refinement of screening and prevention strategies in this population.

A persistent challenge identified was the inconsistent use of terminology. Despite three consensus statements, outdated or imprecise terms remain widespread, which compromises the interpretability and translation of findings [4]. The adoption of anatomy-based classifications, such as those proposed in the Doha agreement, should be prioritised to improve clarity and guide evidence-based management. The Doha agreement represents an international consensus that provides standardised terminology and anatomically based definitions of groin pain in athletes, categorising it into adductor-, iliopsoas-, inguinal-, and pubic-related entities [4].

This review presents some limitations that should be considered when interpreting the findings. A notable gap in the literature is the lack of data on female athletes, despite emerging evidence of sex-specific injury patterns. This underrepresentation may reflect multifactorial barriers, including unconscious referral or recruitment biases, and highlights the need for more inclusive research designs [16]. Additionally, the included studies were heterogeneous in terms of sport types, population characteristics, diagnostic criteria, and outcome definitions, which precluded meta-analytic pooling. Sample sizes varied considerably, and several investigations involved small or homogeneous cohorts, reducing external validity. The overall methodological quality was moderate, with common concerns regarding confounding control and outcome measurement identified through the ROBINS-E assessment. These factors limit the ability to establish causality and generalise the results. Nevertheless, the consistency of associations across studies supports the interpretation that groin pain in athletes arises from a multifactorial interaction between intrinsic and extrinsic risk factors.

By consolidating and critically appraising heterogeneous evidence, this review contributes to establishing a more structured framework for identifying, assessing, and addressing the risk of groin pain in team sport athletes. Although the available evidence remains limited, this review consolidates the most consistent findings and delineates the primary methodological gaps that must be addressed in future research. By integrating recent studies and highlighting the role of subclinical symptoms and sport-specific exposures, it refines the current understanding of groin pain aetiology and provides direction for future preventive approaches. Future studies should adopt prospective designs with standardised diagnostic protocols and robust surveillance systems to enhance the understanding of the disease. Research incorporating biomechanical, neuromuscular, and load-monitoring variables is needed to clarify modifiable risk factors and evaluate the effectiveness of preventive strategies. Consensus-based anatomical classifications and longitudinal follow-ups are crucial for advancing the understanding of groin pain aetiology and prevention.

Clinicians should implement individualised screening programmes that assess hip eccentric adduction strength, hip rotation ROM, and injury history. Practical tools, such as the adductor squeeze test, can help identify at-risk athletes. Preventive programmes should prioritise preseason conditioning, gradual load progression, and ongoing symptom monitoring to maximise performance. Recognising subclinical signs may allow for early intervention and reduce chronic injury development. Beyond isolated screening, effective prevention requires an integrated, multidisciplinary approach involving coaches, strength and conditioning specialists, and medical staff to balance performance goals with injury risk. Education of athletes on early symptom recognition and adherence to preventive exercises should also be emphasised. These clinical strategies, grounded in the current evidence, may support more sustainable participation and performance in team sports.

## 5. Conclusions

This systematic review identified previous groin injury, reduced eccentric hip adduction strength, limited hip rotation range of motion, and inadequate preseason conditioning as the main risk factors for groin pain in team-sport athletes. These findings highlight the multifactorial nature of the condition and support the need for individualised screening and preventive strategies. Despite the moderate methodological quality of the included studies, this review consolidates the current evidence and outlines key priorities for future research, including the development of standardised diagnostic criteria, the use of prospective study designs, and the greater inclusion of female athletes.

## Figures and Tables

**Table 1:** Database search terms and Boolean combinations.

Search Terms	Descriptors
1. Risk Factors	Determinants OR Contributors OR Causes
2. Groin pain	Adductor strain OR Athletic pubalgia OR Inguinal pain OR Pelvic pain
3. Athletes	Sports participants OR Players OR Recreational athletes OR Amateur athletes OR Elite athletes
Combination	

**Table 2:** Eligibility criteria for the inclusion of reviews.

		Inclusion Criteria	Exclusion Criteria
P	Population	Individuals over 18 years of age	-
E	Exposure	Sports in general	-
C	Comparison	-	-
O	Outcome	Risk factors for pubalgia	-
S	Study design	Observational studies (prospective, retrospective, and case–control)	Case reports, commentaries, narrative reviews, and studies not published in peer-reviewed journals

**Table 4:** Risk of bias assessed using the ROBINS-E tool.

Domain	Emery et al., 2001 [24]	Verrall et al., 2007 [27]	Ibrahim, Murrell, and Knapman, 2007 [26]	Hölmich et al., 2014 [17]	Neuville et al., 2023 [28]	Wörner, Thorborg, Clarsen, and Eek, 2022 [29]	Delahunt, Fitzpatrick, and Blake, 2017 [25]	Mosler et al., 2018 [14]
D1—Confounding	Moderate	High	High	Moderate	Moderate	Moderate	Low	Low
Justification	Adjusted for injury history, experience, strength, and flexibility, but residual risk remains.	No adjustment for relevant confounders; limited statistical analysis.	Did not control for important confounders.	Stratified by position and age, but no multivariate adjustment for key confounders.	Adjusted for field position and BMI; residual confounding risk acknowledged.	Adjusted for field position and muscle strength; residual confounding risk acknowledged.	Adjusted for factors such as adductor strength and physical function; relevant confounder control.	Adjusted for eccentric adductor strength and injury history; relevant confounder control.
D2—Exposure Measurement	Low	Moderate	Low	Low	Low	Low	Low	Low
Justification	Prospective measures with valid and reliable instruments.	ROM measured once, no inter/intra-rater reliability, only at 90° flexion.	Hip ROM assessed with goniometer, good intra-rater reliability.	Weekly data collection using standardised questionnaires, ~95% response rate.	OWL exposure recorded throughout the year; reliability not specified.	Isometric hip adduction and abduction measured with handheld dynamometer; reliability not specified.	Adductor squeeze test performed with pressure gauge; reliability not specified.	Musculoskeletal screening tests performed with standardised protocols; reliability not specified.
D3—Participant Selection	Low	Moderate	Moderate	Low	Moderate	Moderate	Low	Low
Justification	Large, representative sample (95% consent rate).	Only 29 of 89 athletes included; exclusion based on subjective criteria.	19 of 120 excluded; justified criteria; included asymptomatic athletes with injury history.	All players from 11 amateur clubs invited and included (n = 998).	1154 university athlete exposures; inclusion criteria not specified.	163 professional and semi-pro ice hockey players; 84% follow-up data provided.	55 Gaelic football players from a single team; homogeneous sample.	438 professional players from Qatar Stars League; 609 player-seasons analysed.
D4—Post-Exposure Interventions	Very Low	Very Low	Very Low	Very Low	Very Low	Very Low	Very Low	Very Low
Justification	No clinical or behavioural interventions after exposure.	No intervention after ROM measurements.	No interventions following ROM measurement.	Observational study, no interventions post-exposure.	No intervention after OWL exposure.	No intervention after OWL exposure.	No intervention after adductor squeeze test exposure.	No intervention after musculoskeletal screening.
D5—Missing Data	Moderate	Low	Low	Moderate	Low	Low	Low	Low
Justification	Some variables not obtained from all (e.g., ~65% reported training).	No follow-up loss reported.	Low and justified losses and exclusions.	Missing data during intermediate weeks, mitigated by intention-to-follow analysis.	Complete data for all exposures; losses not specified.	Complete data for 163 players; losses not specified.	No follow-up loss reported.	No follow-up loss reported.
D6—Outcome Measurement	Low	Low	Low	Moderate	Low	Low	Low	Low
Justification	Injuries recorded in standardised database with clear criteria.	Clearly defined outcome (pain ≥6 weeks + game absence) tracked by physicians.	Standardised clinical definition confirmed with imaging.	Self-reported definition, no clinical/imaging confirmation, reduced precision.	Outcome defined as athletic pubalgia surgery; clinical confirmation not specified.	Hip/groin problems defined as time-loss or non-time-loss; functional assessment not specified.	Groin injuries recorded as time-loss; clinical confirmation not specified.	Groin injuries recorded as time-loss; clinical confirmation not specified.
D7—Selection of the Reported Result	Low	Moderate	Moderate	Low	Low	Low	Low	Low
Justification	Clear results, coherent and no signs of selective reporting.	Limited analysis, partially presented results.	Some relevant analyses not reported; partial presentation.	Results transparently and fully reported.	Complete results consistent with study objectives.	Complete results consistent with study objectives.	Complete results consistent with study objectives.	Complete results consistent with study objectives.
**Overall Judgement**	**Moderate risk of bias**	**Moderate to high risk**	**High risk of bias**	**Moderate risk of bias**	**Moderate risk of bias**	**Moderate risk of bias**	**Low risk of bias**	**Low risk of bias**

Caption: BMI = body mass index; ROM = range of motion; OWL = Olympic weightlifting; n = sample size.

## Data Availability

No new data were created in this study. Data sharing is not applicable to this article.
